# Autophagy suppresses self-renewal ability and tumorigenicity of glioma-initiating cells and promotes Notch1 degradation

**DOI:** 10.1038/s41419-018-0957-3

**Published:** 2018-10-18

**Authors:** Zhennan Tao, Tao Li, Haiwen Ma, Yihan Yang, Chen Zhang, Long Hai, Peidong Liu, Feng Yuan, Jiabo Li, Li Yi, Luqing Tong, Yingshuai Wang, Yang Xie, Haolang Ming, Shengping Yu, Xuejun Yang

**Affiliations:** 1 Department of Neurosurgery, Tianjin Medical University General Hospital, Tianjin, 300052 China; 2 Laboratory of Neuro-Oncology, Tianjin Neurological Institute, Tianjin, 300052 China; 3 Key Laboratory of Post-trauma Neuro-Repair and Regeneration in Central Nervous System, Ministry of Education, Tianjin, 300052 China; 4 Tianjin Key Laboratory of Injuries, Variations and Regeneration of Nervous System, Tianjin, 300052 China; 5 Department of Neuro-Oncology, The University of Texas MD Anderson Cancer Center, Houston, TX 77030 USA; 6 Department of Hematology, Tianjin Medical University General Hospital, Tianjin, 300052 China

## Abstract

Autophagy is a vital process that involves degradation of long-lived proteins and dysfunctional organelles and contributes to cellular metabolism. Glioma-initiating cells (GICs) have the ability to self-renew, differentiate into heterogeneous types of tumor cells, and sustain tumorigenicity; thus, GICs lead to tumor recurrence. Accumulating evidence indicates that autophagy can induce stem cell differentiation and increase the lethality of temozolomide against GICs. However, the mechanism underlying the regulation of GIC self-renewal by autophagy remains uncharacterized. In the present study, autophagy induced by AZD8055 and rapamycin treatment suppressed GIC self-renewal in vitro. We found that autophagy inhibited Notch1 pathway activation. Moreover, autophagy activated Notch1 degradation, which is associated with maintenance of the self-renewal ability of GICs. Furthermore, autophagy abolished the tumorigenicity of CD133 + U87-MG neurosphere cells in an intracranial model. These findings suggest that autophagy regulating GICs self-renewal and tumorigenicity is probably bound up with Notch1 degradation. The results of this study could aid in the design of autophagy-based clinical trials for glioma treatments, which may be of great value.

## Introduction

Glioblastomas (GBMs) are the most common and lethal primary central nervous system tumors and have a poor prognosis^1,2^. The current standard-of-care treatment consists of maximal surgical resection followed by radiotherapy and subsequent temozolomide treatment. Even with advances in targeted therapies and immunotherapies, the median survival duration of GBM patients is only 14.6 months^3^. Glioma stem cells, i.e., glioma-initiating cells (GICs), which are capable of self-renewal, infinite proliferation, multiple potential differentiation, and vigorous tumorigenicity, are closely associated with GBM resistance to chemotherapy and radiotherapy^4,5^.

The Notch pathway is important in the maintenance of GIC self-renewal and tumorigenicity^6^, and the GIC population increases as a result of Notch pathway activation^7^. Our previous study characterized the Notch1 pathway mediated maintenance of the stem cell phenotype in GBMs^8^. Four Notch receptors (Notch1–4) and five Notch ligands (Jagged-1 and 2 and Delta-like-1, 3, and 4) have been identified in mammals^9^. The Notch pathway is triggered when a Notch ligand binds to a Notch receptor on a neighboring cell; this binding leads to proteolytic cleavage of the Notch receptor and endocytosis of the Notch extracellular domain into the signal-sending cell^10^. The Notch intracellular domain (NICD) is then released, translocates into the nucleus, and interacts with the CBF1/RBP-Jκ/Suppressor of Hairless/LAG-1 complex to trigger a cascade of events that leads to the upregulation of Hes and Hey family genes^11^. Although early therapy targeting the Notch pathway can suppress the formation of a hypoxic tumor microenvironment and promote cell apoptosis, it has no significant benefit for GBM patients undergoing long term treatment^12–16^. The novel mechanism underlying Notch-pathway-dependent therapy will be discussed in this study.

Autophagy is an evolutionarily conserved lysosome-dependent process that involves degradation of long-lived proteins and dysfunctional organelles and contributes to cell metabolism^17^. In cancers, autophagy has pivotal functions since it prevents tumor progression^18–20^. Recently, autophagy has been shown to promote differentiation and attenuate self-renewal of GICs^21,22^. However, how autophagy regulates differentiation and self-renewal of GICs is not well understood.

Autophagy is associated with the Notch pathway. In biliary differentiation, the loss of autophagy leads to activation of the Notch pathway^23^. During bone marrow mesenchymal stem cell proliferation, autophagy inhibits the Notch1 pathway, thus, suppressing cell proliferation^24^. Therefore, we speculate that autophagy and the Notch1 pathway may be related with respect to regulation of GIC self-renewal. However, the mechanism underlying regulation is not known.

In the present study, we evaluated the association between autophagy and the Notch1 pathway in the context of GIC self-renewal. Our findings for the first time have shown that autophagy suppressed GIC self-renewal and tumorigenicity. Also, our data reveal that autophagy inhibit Notch1 pathway activation by upregulating Notch1 degradation. Therefore, autophagy-induced Notch1 degradation could be a promising treatment strategy for preventing GBM progression.

## Results

### CD133 + glioma neurospheres exhibited high Notch pathway activity

To investigate the mechanism underlying maintenance of stemness in GICs, we established a CD133 + glioma neurosphere model in vitro. First, magnetic-activated cell sorting (MACS) was used to collect CD133 + cells from U87 and U251 glioma cells. Flow cytometry was then performed to quantify the CD133 + cells in the MACS + population to confirm the effectiveness of the sorting. Prior to performing MACS, CD133 + cells constituted only 6.65 ± 0.6% of U87-MG and 5.98 ± 0.93% of U251-MG cells. After sorting, the percentage of CD133 + cells (87.64 ± 4.09% in U87-MG and 76.93 ± 3.59% in U251-MG) was significantly higher (Supp. Fig. 1a). The CD133 + cells were then cultured in stem cell medium and formed neurospheres, while cells not sorted on the basis of CD133 positivity failed to develop spheroids under the same culture conditions (Supp. Fig. 1b). The resulting neurospheres were used in the subsequent studies.

CD133 and Nestin were selected as markers to assess GIC stemness. Notch1 pathway activation was evaluated based on expression of NICD and target gene HES1. Cell differentiation was assessed by expression of the astrocyte marker, glial fibrillary acidic protein (GFAP). CD133 + neurospheres exhibited higher expression of stem cell markers (CD133 and Nestin), Notch1, and activated Notch1 pathway-related proteins (NICD and HES1) than pre-MACS cells as shown by western blot. Moreover, there was decreased expression of GFAP in CD133 + neurospheres (Supp. Fig. 1c). Immunofluorescence staining revealed that stem cell markers (CD133 and Nestin), Notch1, and NICD were strongly expressed in CD133 + neurospheres (Supp. Fig. 1d). These results showed that Notch1 pathway activation was elevated in CD133 + glioma neurospheres.

### AZD8055 and rapamycin-induced autophagy in GICs

To investigate whether autophagy in GICs is induced by inhibition of mTOR signaling, AZD8055 and rapamycin were used. Signaling through mTOR regulates many fundamental cell processes, including autophagy^25,26^. The mTOR complex 1 (mTORC1) can actively suppress autophagy by phosphorylating Unc-51-like autophagy activating kinase 1 (ULK1)^27^. Activation of key autophagy genes, such as LC3B, Gabarap, ULK2, and Beclin-1, is inhibited by mTOR complex 2 (mTORC2) through AKT Ser473 phosphorylation^28^. Rapamycin, which is a typical mTORC1 inhibitor, is a powerful autophagy inducer^29^. AZD8055, a new ATP-competitive mTOR kinase inhibitor, can potently and selectively block both mTORC1 and mTORC2 activity. It is a stronger inducer of autophagy than rapamycin^28^.

**Fig. 1:**
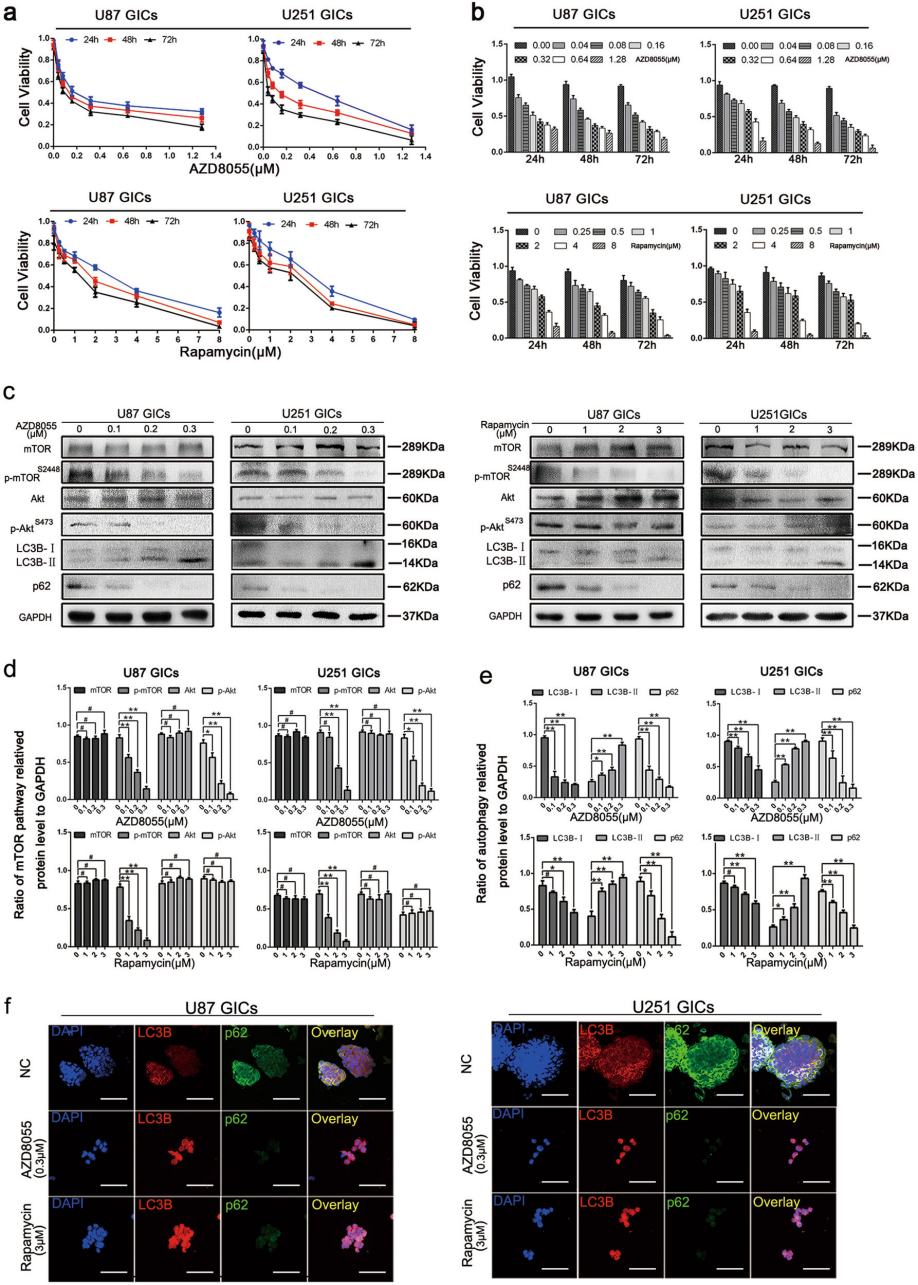
AZD8055 and rapamycin inhibited mTOR signaling pathway followed by inducing autophagy. **a**, **b** U87 and U251 GICs were treated with AZD8055 and rapamycin for 24, 48, 72, and cell viability was detected using CCK-8 assay. **c**–**e** U87 and U251 GICs were treated with various concentrations of AZD8055 for 48 h and rapamycin for 24 h. The cells were harvested, and the effects of AZD8055 and rapamycin on the protein expression of the mTOR pathway and autophagy-related were detected by western blot. Data are shown as means ± s.d., *n* = 3, ^#^*P* = NS, *P < 0.05, **P < 0.01, Student’s *t*-test. **f** Immunofluorescence staining of U87 and U251 GICs, which treated by DMSO, AZD8055 (0.3 μM) and rapamycin (3 μM). The nuclei were stained with DAPI and the antibody against LC3B and p62. Images were captured by laser confocal microscope ( × 400), scale bar = 100 μm

Next, the effects of rapamycin and AZD8055 on GIC viability were assessed using the Cell Counting Kit-8. Treatment with different concentrations of AZD8055 and rapamycin for different durations decreased cell viability (Fig. 1a, b). Preliminary experiments revealed effective durations of treatment for AZD8055 and rapamycin were 48 and 24 h, respectively, in U87 and U251 GICs. The IC50 values for 48 h of AZD8055 treatment were approximately 0.27 μM in U87 and 0.18 μM in U251 cells and for 24 h of rapamycin treatment were 2.7 μM in U87 and 3 μM in U251 cells. Based on the IC50 values, the treatment concentrations used for the subsequent experiments were 0.1, 0.2, and 0.3 μM for AZD8055 when treating U87 and U251 cells for 48 h and 1, 2, and 3 μM for rapamycin when treating U87 and U251 cells for 24 h.

Previous studies indicate mTORC1 primarily contains mTOR with phosphorylated Ser2448 and mTORC2 contains mTOR with phosphorylated Ser2481^30^. To regulate protein translation, mTORC1 phosphorylates p70S6K and 4E-BP1^31^. To enhance Akt enzymatic activity, mTORC2 phosphorylates Ser473 of Akt^32^. To confirm mTOR signaling is inhibited on treatment with rapamycin and AZD8055, the levels of mTOR pathway-related proteins, mTOR, p-mTOR (Ser2448), Akt, and p-Akt (Ser473) were measured by western blotting. AZD8055 and rapamycin both suppressed p-mTOR (Ser2448) expression, but only AZD8055, not rapamycin, inhibited p-AKT (Ser473). These results indicate that both AZD8055 and rapamycin can suppress the mTOR pathway in GICs; rapamycin inhibits mTORC1 only and AZD8055 inhibits both mTORC1 and mTORC2 (Fig. 1c, d).

It is well known that the mTOR signaling pathway can induce autophagy by modulating the formation of the downstream Autophagy-related 1 (Atg1)/ULK1 complex^33^. The effect of AZD8055 and rapamycin on the induction of autophagy was evaluated by western blot and immunofluorescence staining. LC3BI-II conversion and downregulation of the expression of the ubiquitin-binding protein p62/sequestosome 1 were used to verify autophagy induction^34^. LC3B increased and p62 decreased in a dose-dependent manner as measured by western blot and immunofluorescence staining following AZD8055 and rapamycin treatment (Fig. 1c, e). Autophagy in U87 and U251 cells was significantly increased by treatment with 0.3 μM AZD8055 and 3 μM rapamycin compared to the control. These results indicate that autophagy can be induced in GICs by AZD8055 and rapamycin treatment.

### AZD8055- and rapamycin-induced autophagy inhibited GIC self-renewal and proliferation in vitro

**Fig. 2:**
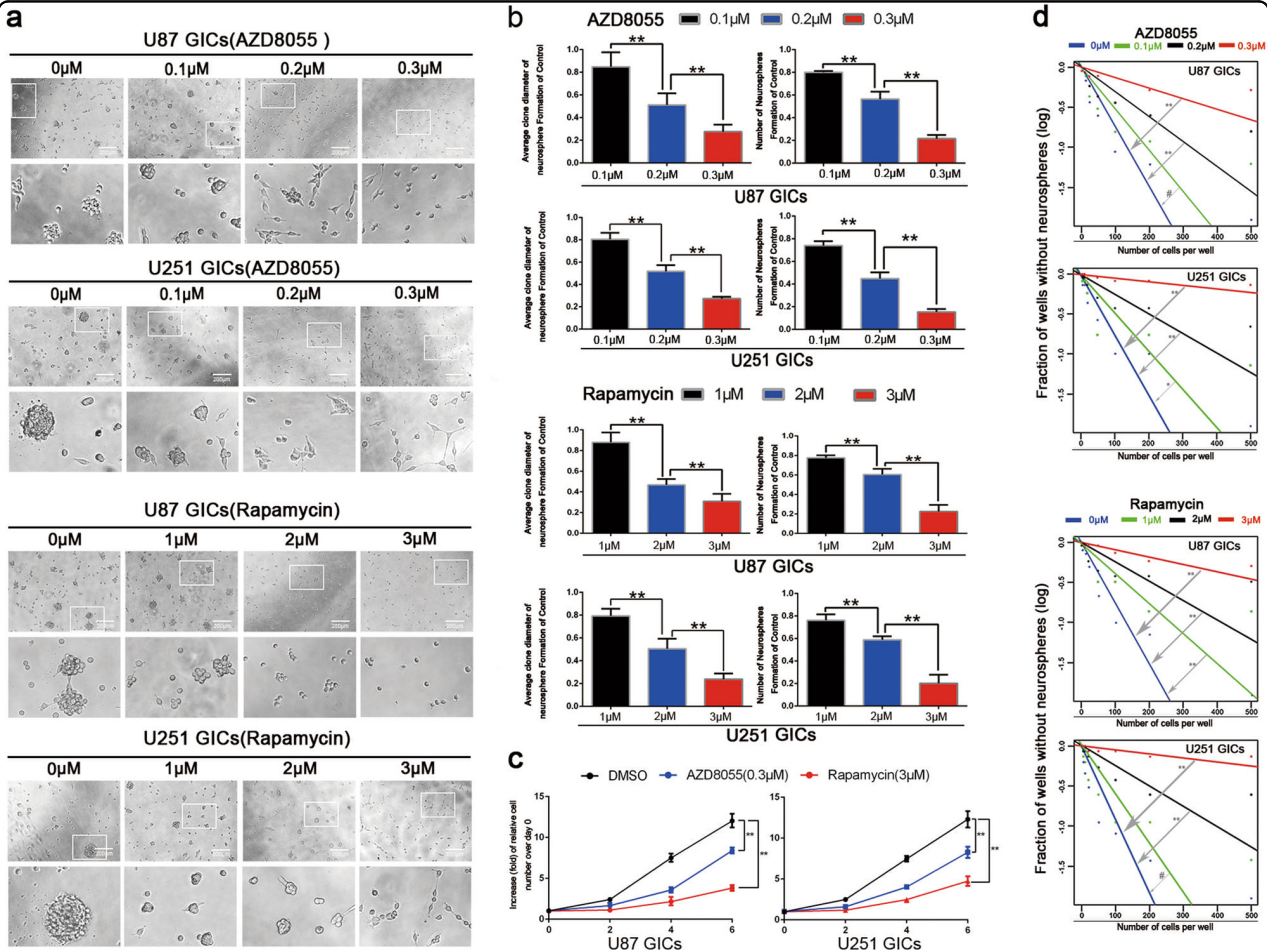
GICs treated with AZD8055 and rapamycin showing that autophagy suppressed GICs proliferation and self-renewal. **a** The representative images of GICs neurospheres showed that neurosphere formation ability of GICs was significantly inhibited by AZD8055 and rapamycin treatment. *n* = 5, Scale bar = 200 µm. **b** The quantification of numbers and diameter of the GICs neurospheres showing that neurosphere formation ability of GICs were obviously inhibited after AZD8055 and rapamycin treatment. **c** The ability of GICs proliferation were showed by cell viability assay. **d** The ability of GICs self-renewal was detected by in vitro limiting dilution assay. Data are shown as means ± s.d, *n* = 5, ^#^*P* = NS, *P < 0.05, **P < 0.01, likelihood ratio test. Data in **b**, **c** are shown as means ± s.d., *n* = 5, *P  < 0.05, **P  < 0.01, Student’s *t*-test

To clarify whether autophagy inhibits GIC self-renewal and proliferation, limiting dilution, neurosphere formation, and cell viability assays were performed^35^. The neurosphere formation assay revealed AZD8055 and rapamycin effectively blocked GIC neurosphere formation (Fig. 2a). By quantifying and measuring the diameter of the neurospheres from U87 and U251 cells, we confirmed the effectiveness of treatment with 0.3 μM AZD8055 and 3 μM rapamycin (Fig. 2b). The cell viability assays determined AZD8055 and rapamycin decreased GIC proliferation (Fig. 2c). For limiting dilution assays, the fraction of wells lacking neurospheres increased gradually as the concentrations of AZD8055 and rapamycin increased. Treatment with 0.3 μM AZD8055 and 3 μM rapamycin significantly reduced the self-renewal of U87 and U251 GICs compared to the control group (Fig. 2d).

**Fig. 3:**
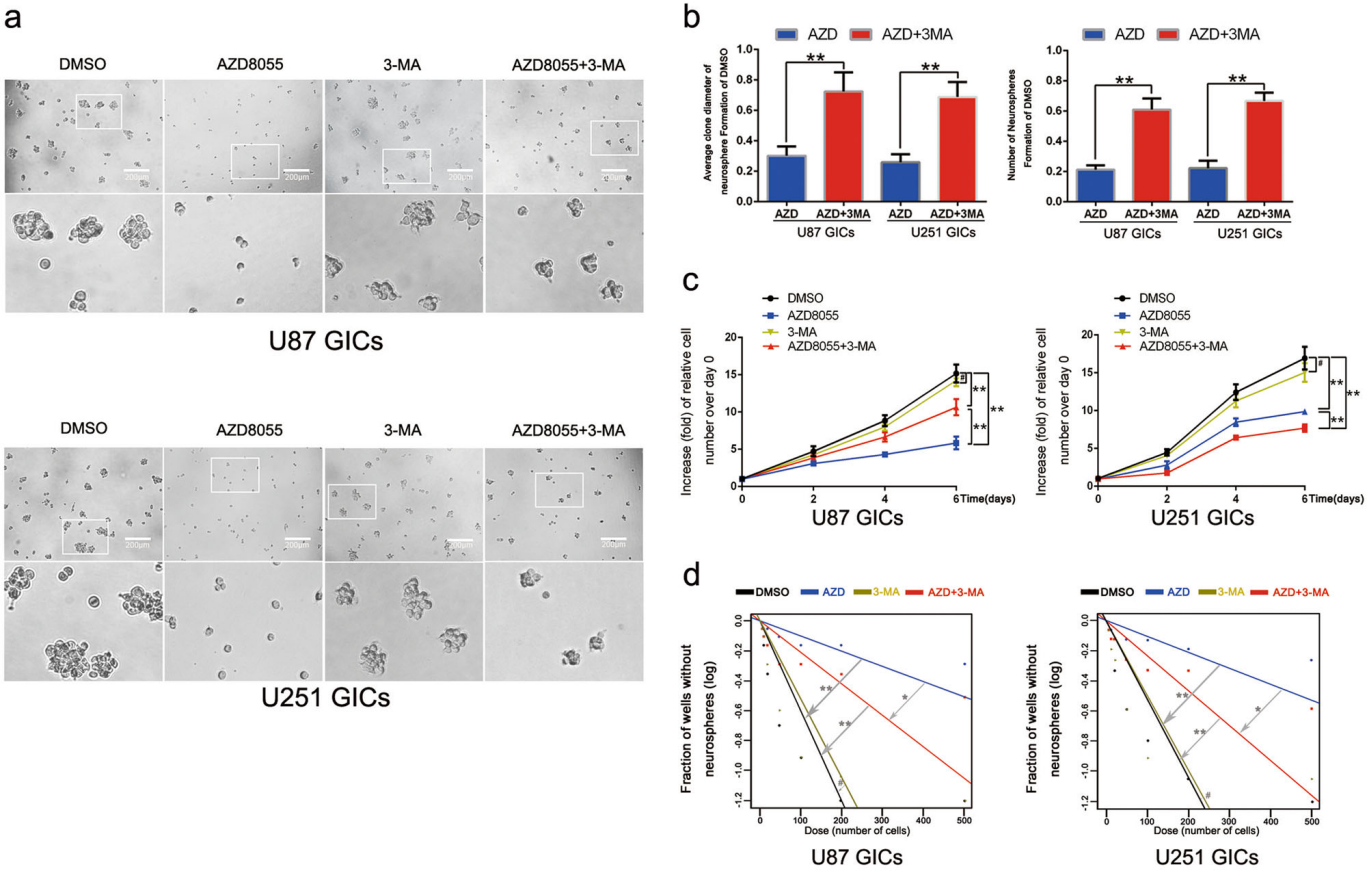
3-MA counteracted the suppression of AZD8055 on GICs self-renewal and proliferation. **a** The representative images of GICs neurospheres showed that self-renewal ability of GICs was significantly inhibited by DMSO, AZD8055, and AZD8055 combined with 3-MA treatment. *n* = 5, Scale bar = 200 µm. **b** The quantification of numbers and diameter of the GICs neurospheres showed that neurosphere formation ability of GICs was rescued after 3-MA treatment. **c** The ability of GICs proliferation was detected by cell viability assay. **d** The ability of GICs self-renewal were detected by in vitro limiting dilution assay. Data are shown as means ± s.d, *n* = 5, *P < 0.05, **P < 0.01, likelihood ratio test. Data in **b**, **c** are shown as means ± s.d., *n* = 5, *P  < 0.05, **P < 0.01, Student’s *t*-test

To further verify that whether the GIC self-renewal impairment is caused by autophagy rather than by mTOR pathway inhibition, we introduced 3-methyladenine (3-MA), an early stage autophagy inhibitor^36^, is widely studied in cancers^37,38^. In this part, 3-MA concentration is 0.5 mM, as a low concentration maintaining about 85% cells survival (Supp. Fig. 2a, b). GICs were treated with DMSO, AZD8055, 3-MA, and AZD8055 + 3-MA combination, respectively. In western blot, changes of LC3B expression showed that 3-MA with 0.5 mM inhibited AZD8055-induced autophagy (Supp. Fig. 2c, d). The neurosphere formation assay revealed that AZD8055 suppressed GICs neurosphere formation. Intriguingly, after combined treatment with 3-MA, neurosphere disruption was reversed (Fig. 3a). By quantifying and measuring the diameter of the neurospheres from U87 and U251 cells, we measured the effectiveness of AZD8055 with or without 0.5 mM 3-MA (Fig. 3b). What’s more, 3-MA showed an antagonistic effect on AZD8055-mediated reduction of GICs proliferation (Fig. 3c). For limiting dilution assays, similarly, AZD8055 + 3-MA treatment decreased the fraction of wells without neurosphere much more than AZD8055 treatment alone (Fig. 3d). These results confirmed that autophagy directly suppressed GIC self-renewal capacity.

**Fig. 4:**
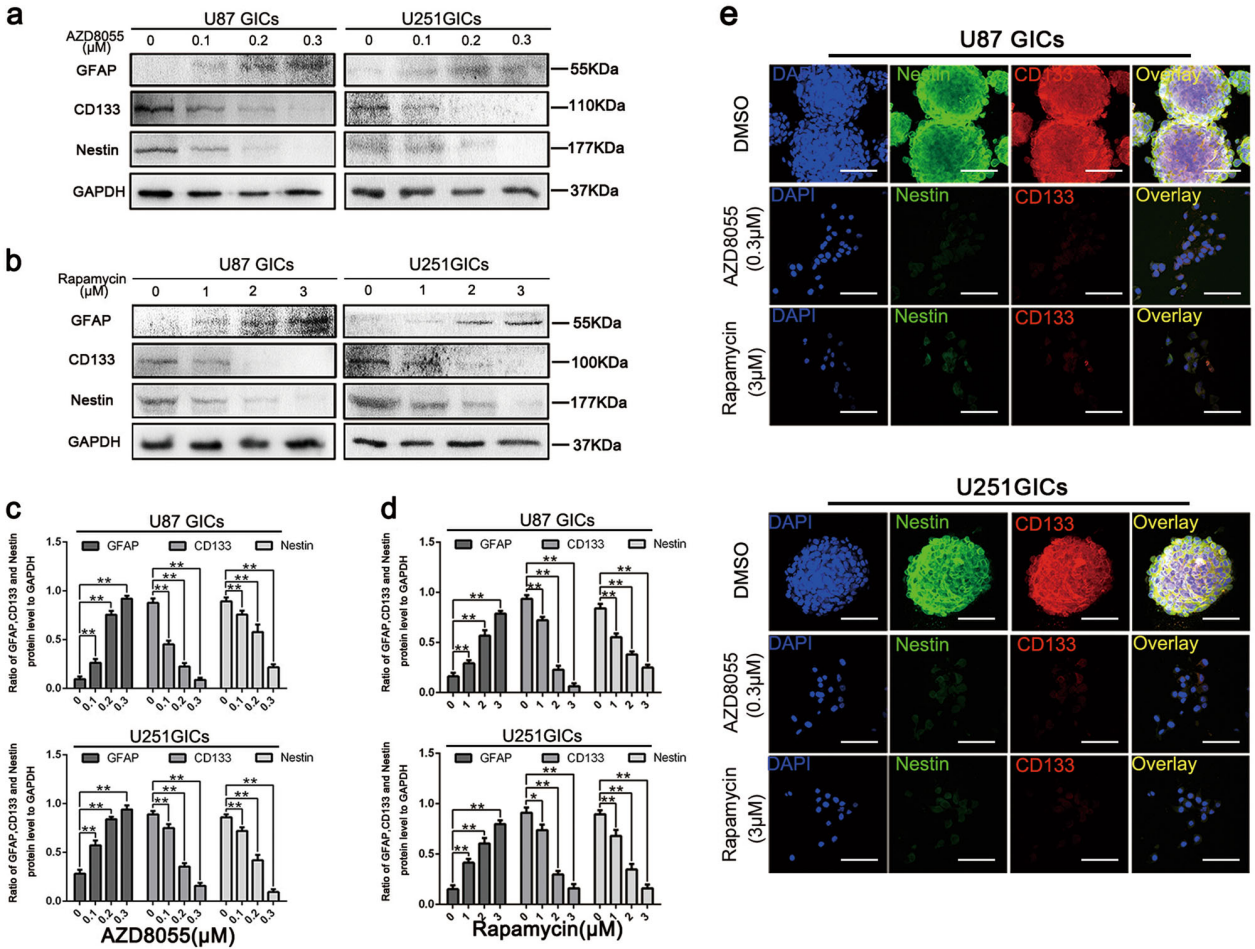
Autophagy effectively downregulated the expression of CD133, Nestin, and GFAP. **a**–**d** U87 and U251 cells were treated the same as in (Picture 2). Protein expressions of CD133, Nestin, and GFAP were detect by western blot. Data are shown as means ± s.d., *n* = 3, *P < 0.05, **P < 0.01, Student’s *t*-test. **e** Immunofluorescence staining of U87 and U251 GICs, which treated by DMSO, AZD8055 (0.3 μM), and rapamycin (3 μM). The nuclei were stained with DAPI and the antibody against CD133 and nestin. Images were captured by laser confocal microscope ( × 400), scale bar = 100 μm

The expression of stem cell markers, CD133 and Nestin, and cell differentiation marker, GFAP, was also measured in U87 and U251 GICs. AZD8055 and rapamycin treatments decreased Nestin and CD133 and increased GFAP expressions at protein level (Fig. 4a–e). Treatment with 0.3 μM AZD8055 and 3 μM rapamycin significantly suppressed GIC stemness maintenance as illustrated by western blot and immunofluorescence staining. These results reveal that AZD8055 and rapamycin-induced-autophagy suppressed self-renewal and proliferation of U87 and U251 neurospheres.

### AZD8055 and rapamycin abolished Notch1 activity

**Fig. 5:**
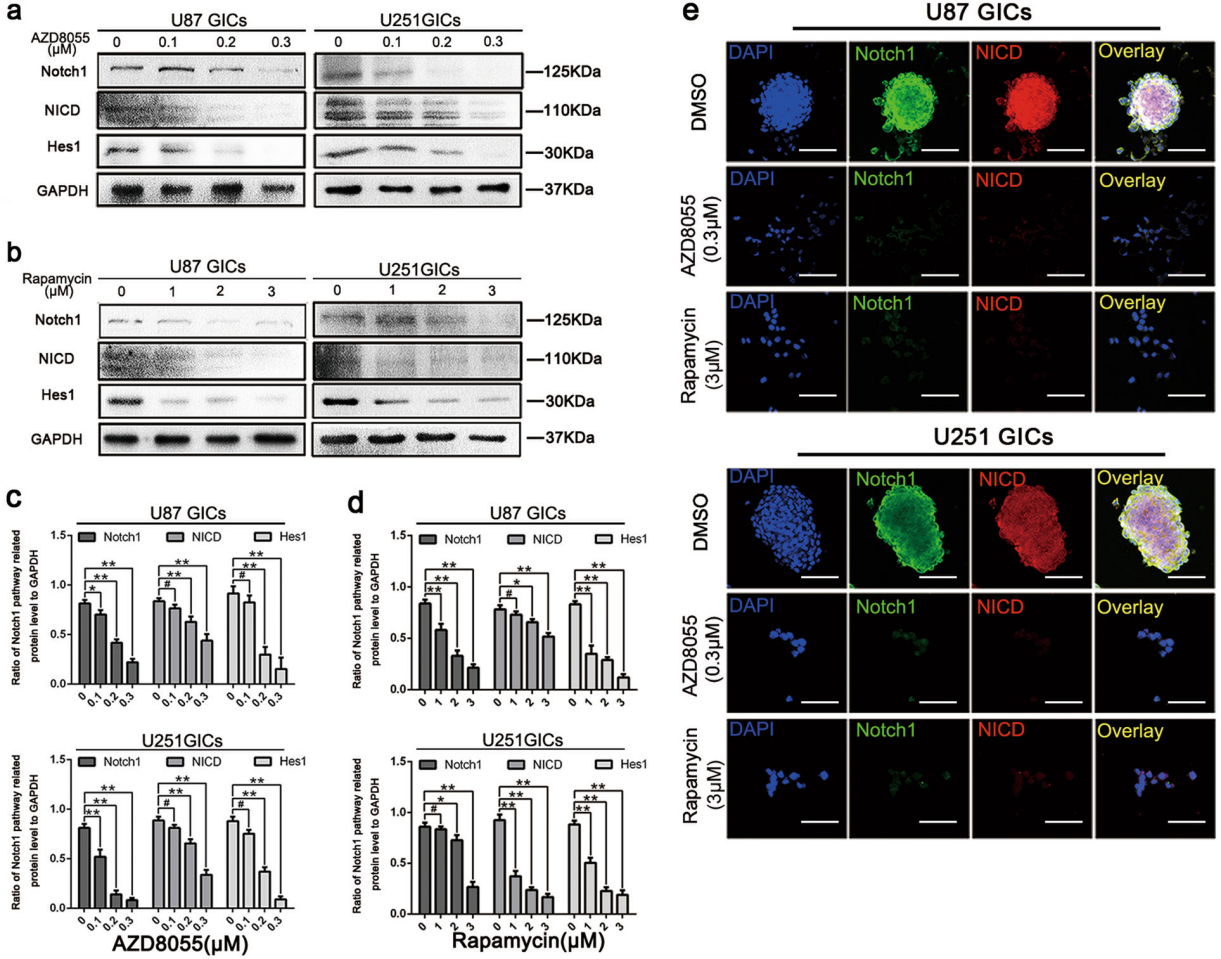
Autophagy reduced the protein expression of Notch pathway. **a**–**d** U87 and U251 cells were treated the same as in (Picture 2). Protein expressions of Notch1 pathway were detect by western blot. Data are shown as means ± s.d., *n* = 3, ^#^*P* = NS, *P < 0.05, **P < 0.01, Student’s *t*-test. **e** Immunofluorescence staining of U87 and U251 GICs, which treated by DMSO, AZD8055 (0.3 μM) and rapamycin (3 μM). The nuclei were stained with DAPI and the antibody against Notch1 and NICD. Images were captured by laser confocal microscope ( × 400), scale bar = 100 μm

Based on the observations described above, GIC self-renewal and proliferation was found to be inhibited by autophagy. To investigate the potential underlying mechanism, close attention was paid to the Notch1 pathway, which is considered a pivotal regulator of maintenance of the GIC stem cell phenotype^6,8^. In this present study, it has been shown that Notch pathway activation leads to NICD translocation to the nucleus and promotes transcription of Hes1. Here, we showed that AZD8055 and rapamycin downregulated Notch1, NICD, and Hes1 expressions significantly in a dose-dependent manner (Fig. 5a–d). Immunofluorescence staining revealed that Notch1 and NICD expression in GICs was abrogated after AZD8055 or rapamycin treatment (Fig. 5e). These results suggested that AZD8055 and rapamycin decreased Notch1 pathway activation.

### AZD8055- and rapamycin-induced autophagy regulated Notch1 degradation

**Fig. 6:**
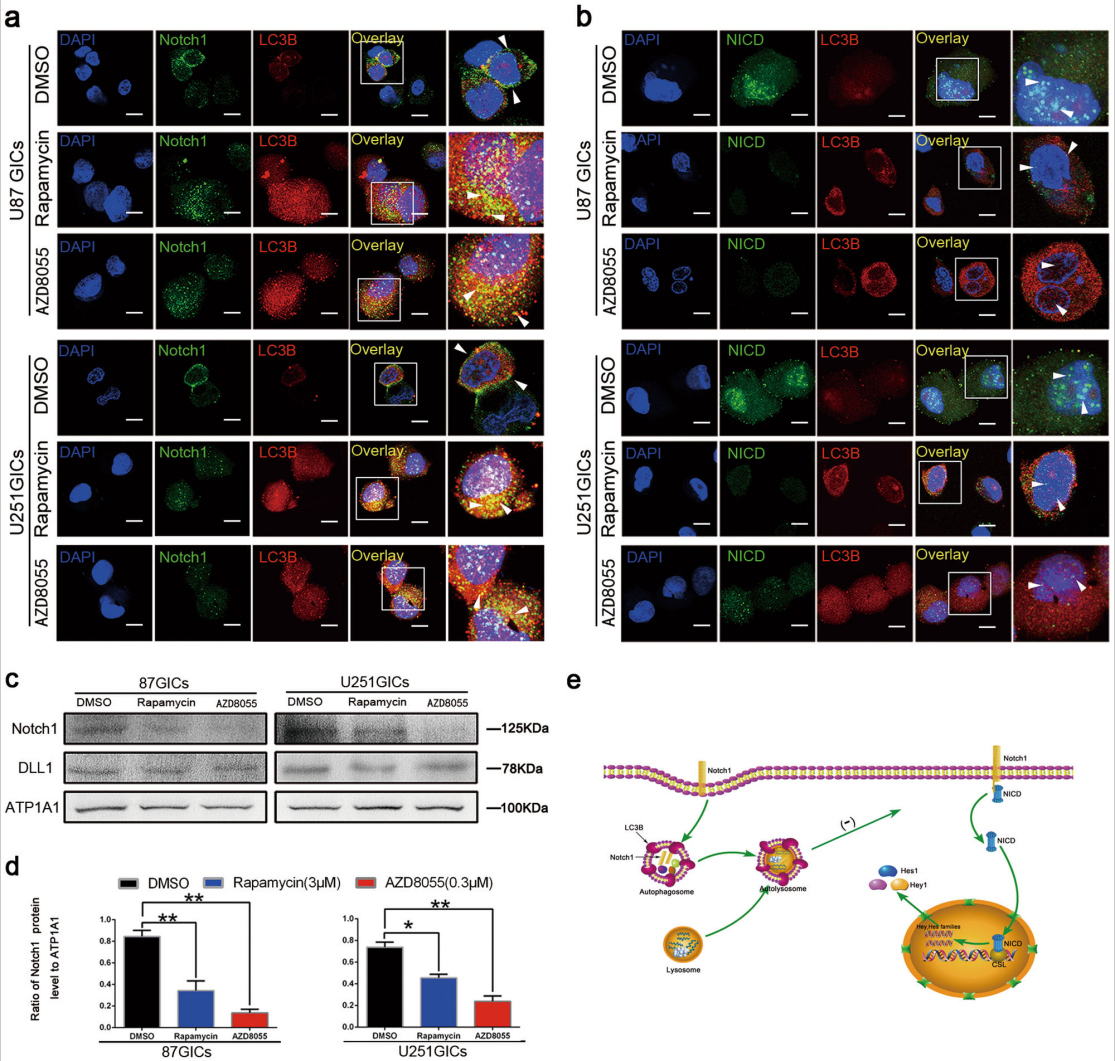
Autophagy regulated Notch1 degradation. **a** U87 and U251 GICs were treated with AZD8055 (0.3 µM) for 48 h and rapamycin (3 µM) for 24 h. Cells were immunostained for Notch1 (green) and LC3 (red) and stained with DAPI (blue). The white box indicates the location of the enlargement. The white triangle shows LC3B and Notch1 colocalization with some yellow points. Scale bar = 5 µm. **b** Cells were immunostained for NICD (green) and LC3B (red) and stained with DAPI (blue) after the same treatment as **a**. Scale bar = 5 µm. **c** The expression of Notch1, DLL1 on the membrane of U87 and U251 GICs were detected by western blot. Drugs treatment were the same as in **a**. **d** Bar chart were used to compare the expression of Notch1 in three groups treatment. Data are shown as means ± S.D. *n* = 3, *P < 0.05, **P < 0.01, Student’s *t*-test. **e** Mechanism diagram described the line of Notch pathway activation and the progress of Autophagy regulating Notch1 degradation

When characterizing the mechanism underlying the influence of autophagy on Notch1 levels and pathway activation, we first discovered that autophagy induced by AZD8055 and rapamycin, can regulate Notch1 degradation. We confirmed that autophagy impacted plasma membrane Notch1 levels, where Notch1 can bind to its cognate ligand and become activated. Both AZD8055 and rapamycin treatments decreased Notch1 subcellular localization on cell membrane (Fig. 6c, d). Theoretically, Notch1 should enter into autophagosomes, which co-localize with LC3B, following autophagic degradation. Based on immunofluorescence staining, Notch1 was clearly localized to the plasma membrane of DMSO-treated groups, while a small fraction of Notch1 co-localized with LC3B in the AZD8055- and rapamycin-treated groups (Fig. 6a). Moreover, Dll1 levels did not change upon induction of autophagy as measured by western blot (Fig. 6c). Furthermore, NICD did not co-localize with LC3B after AZD8055 and rapamycin treatment as determined by immunofluorescence staining (Fig. 6b). This indicated that the decreased levels of NICD was a consequence of Notch1 degradation rather than NICD degradation. These results are presented in a mechanistic diagram showing that autophagy suppressed Notch1 pathway activity through inducing Notch1 degradation (Fig. 6e).

### AZD8055 and rapamycin suppressed GIC tumorigenicity in intracranial xenograft model

**Fig. 7:**
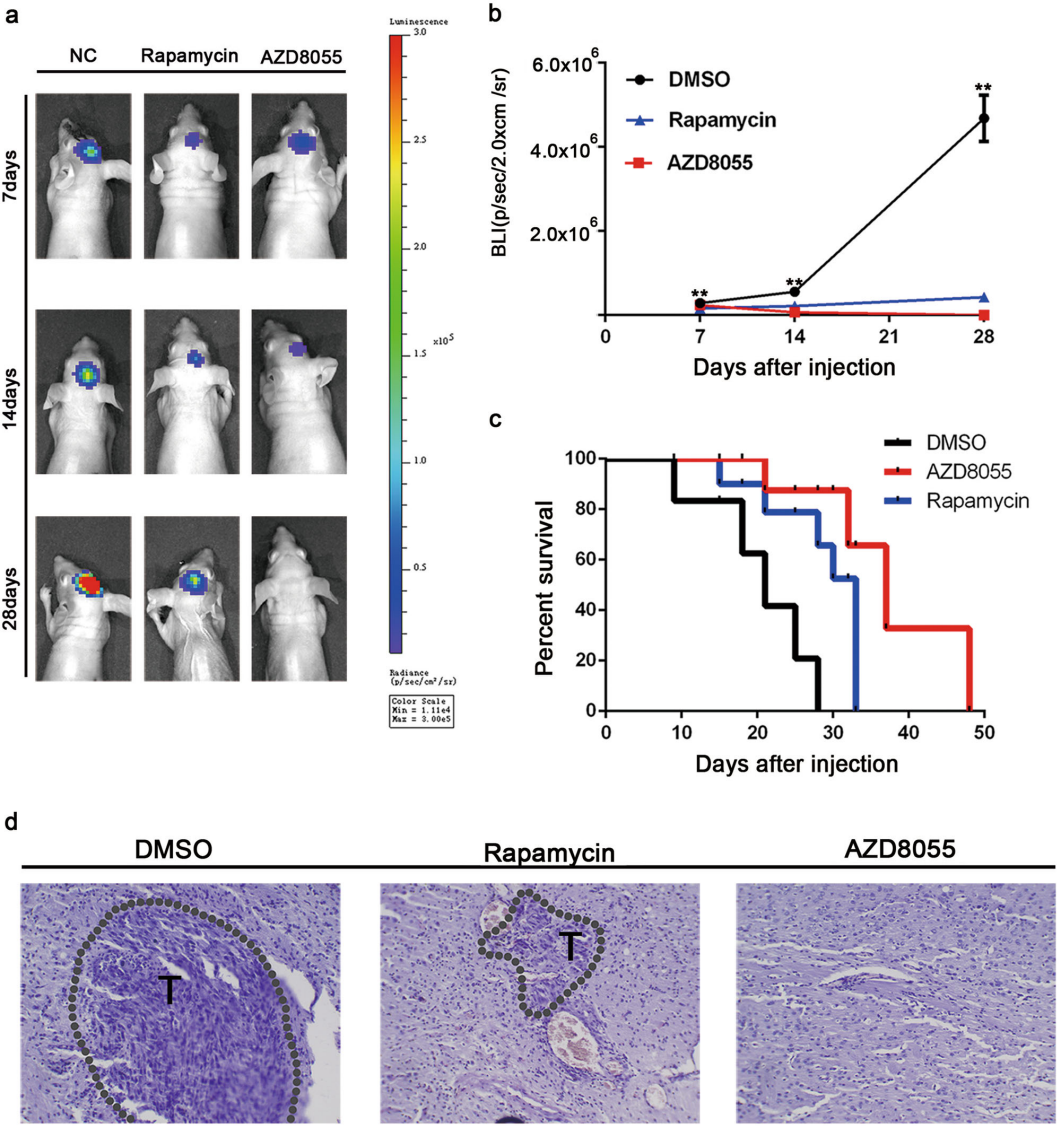
AZD8055 and rapamycin inhibited the tumorigenicity of CD133 + U87-MG neurosphere cells in vivo. The mice were treated with intraperitoneal injection with DMSO, AZD855 (10 mg/kg/day), or rapamycin (1.5 mg/kg/day) for 5 days a week. The treatment started from the 7th day after implantation and lasted for ~21 days. **a** Representative images of bioluminescence of mice on days 7, 14, and 28 after implantation. **b** Quantitative analysis of these bioluminescence images for the DMSO, AZD8055, and rapamycin treatment groups. **c** The overall survival of mice in the DMSO, AZD8055, and rapamycin treatment groups. Data are shown as the mean ± S.D. *n* = 5, **P < 0.01 compared to the control, ANOVA test. **d** Representative images of the HE staining in tumor sections ( × 400 magnification)

In vivo, tumor volume is commonly utilized to evaluate GIC tumorigenicity. To investigate the potential effects of AZD8055 and rapamycin on GIC tumorigenicity, an intracranial orthotopic xenograft model was generated by implanting U87 GICs into the brain. Seven days after implantation, DMSO, AZD8055, or rapamycin was injected intraperitoneally 5 days a week for 3 weeks. Bioluminescence imaging and hematoxylin-eosin staining revealed that GIC tumorigenicity was lower in the AZD8055 and rapamycin groups than that in the DMSO group; in particular, AZD8055 treatment resulted in smaller tumor volumes than rapamycin treatment (Fig. 7a, b, and d). AZD8055- and rapamycin-treated mice survived markedly longer than DMSO-treated mice, where AZD8055-treated mice harbored the longest survival among the cohorts (Fig. 7c). These results show that AZD8055- and rapamycin-induced autophagy suppressed tumorigenicity of U87 GICs in vivo.

## Discussion

In the present study, we found that autophagy suppressed GIC self-renewal and tumorigenicity in vitro and in vivo and promoted Notch1 degradation, thus, inhibiting Notch1 pathway activation. These findings reveal a novel approach to prevent GBM recurrence by targeting GICs. Therefore, we detected a stemness-destructed autophagy induced by mTOR inhibitors, which is supported by previous studies^23,24^. Although other studies suggests that autophagy is protective^39,40^, we argue that tumor cells have to derive adequate nutrient substance to guarantee basic survival-related sustainment upon various stresses by sacrificing some aggressive behaviors, such as stemness maintenance. Similarly, we detected that mTOR inhibitors, an autophagy inducer, exerted an effect of suppression on the self-renewal and tumorigenicity of GICs and could be blocked by using autophagy inhibitors in vitro.

GICs perform an important biological role in the cell population. The key features distinguishing GICs from other tumor cells include their abilities to self-renew, differentiate into heterogeneous types of tumor cells, and sustain tumor growth in vivo^41^. There has been increasing evidence that GICs are responsible for tumor initiation, resistance to current therapies, and tumor recurrence^42–44^. The mechanisms underlying GIC self-renewal and tumorigenicity have long been a conundrum for researchers^45,46^. In the present study, the expression of stem cell markers, CD133 and Nestin, confirmed that the cells collected by MACS were a highly pure population of GICs. The modus operandi has been described in our previous study^8^. In addition, a recent study has shown the transcription factors OCT4, SOX2, KLF4, and MYC can also be regarded as stem cell markers and play a role in regulation of self-renewal, pluripotency, and tumorigenicity of gliomas^47^.

There are several methods to induce autophagy, including hypoxia, starvation, and Beclin-1 activation. In our study, autophagy was induced by treatment with AZD8055 and rapamycin. Using this model, we found that the self-renewal and proliferation of GICs was suppressed by AZD8055 and rapamycin treatment as shown by limiting dilution, neurosphere formation, and cell viability assays. Changes in CD133, Nestin, and GFAP protein levels also supported a decrease in GIC self-renewal ability. These results support past studies showing that autophagy can impact differentiation in human tendon stem and hematopoietic stem cells^22,48,49^. In addition, we confirm the direct influence of autophagy on the self-renewal capacity of GICs by using 3-MA. Although some literatures suggested that 3-MA potentiated tumor cell apoptosis and enhanced the effect of chemotherapy sensitivity^50,51^, our data revealed that 3-MA inhibited AZD8055-induced autophagy and rescued AZD8055-induced GICs self-renewal and proliferation inhibition. Similarly, several studies showed that inhibition of autophagy by 3-MA partially inhibited AZD8055-induced cell death^38^.

Moreover, our study suggests that induction of autophagy by AZD8055 and rapamycin can attenuate the activity of the Notch pathway. In gliomas, the Notch pathway has been shown to maintain the stem cell phenotype. In the Notch pathway, Notch1 is the most important molecule that is involved in tumor cell proliferation, apoptosis, and invasion and plays a major role in stem cell self-renewal, migration, and other biological behaviors^10,52^. In this study, autophagy was found to suppress Notch1 pathway activation. These results are consistent with previous studies reporting that loss of autophagy can lead to the activation of Notch signaling during oogenesis in Drosophila^53^ and regulate biliary differentiation of hepatic progenitor cells through the Notch1 pathway^23^.

Furthermore, autophagy impacts the Notch1 pathway by upregulating Notch1 degradation. These results suggest that Notch1 expression on the cell membrane is decreased and autophagy-induced Notch pathway inhibition is Notch1 ligand, DLL1- and NICD-independent. Immunofluorescence staining also provided evidence of Notch1 entering autophagic vesicles. The above results are all in accordance with those of the study conducted by Wu et al.^54^. Wu’s study showed that autophagy regulates Notch1 degradation; this degradation correlates with the expected effect of Notch hyperactivity on stem cell development and neurogenesis. It is worth noting that studies conducted by other groups have found that autophagy can eliminate cytoplasmic NICD, thus, regulating breast cancer tumor cell proliferation^17^. However, the data obtained in this study does not explain how Notch1 enters the autophagosome. Presently, there are two predominant hypotheses explaining this process. One possibility is that Notch1 can enter autophagosomes in an endocytosis-independent manner^55,56^. In contrast, Notch1 could be transported to autophagosomes from the plasma membrane via precursor autophagic vesicles^54^. Therefore, further studies are needed to characterize the mechanism underlying autophagy-mediated regulation of Notch1 degradation.

Finally, in intracranial xenograft models, autophagy inhibited GIC tumorigenicity. The mTOR inhibitors, in particular, AZD8055, not only significantly suppressed brain tumor growth but also prolonged the integrity of the intracranial blood brain barrier. Similarly, a recent study showed that PI3K/mTOR-inhibitors with pronounced brain penetration inhibited brains tumors in a targeted manner in Abcb1a/b-/- and Abcg2-/- mice^57^.

In conclusion, the results of this present study support a novel glioma therapeutic strategy based on inhibiting GIC self-renewal and tumorigenicity. Inducing autophagy to suppress Notch1 pathway activation may be a promising approach for inhibiting GICs. Therefore, studies on the molecular targets and/or signaling pathways that induce autophagy and, thus, regulate Notch1 degradation must be conducted in the future as they may provide a novel basis for treating GBMs.

## Materials and methods

### Antibodies

Antibodies against Nestin (Ab11306; dilution for western blotting, 1:1000; dilution for immunofluorescence analysis, 1:100), CD133 (Ab19898; dilution for western blotting, 1:1000; dilution for immunofluorescence analysis, 1:100), Notch1 (Ab52627/Ab44986; dilution for western blotting, 1:1000; dilution for immunofluorescence analysis, 1:100), NICD (Ab8925; dilution for western blotting, 1:1000; dilution for immunofluorescence analysis, 1:100), Hes1 (Ab71559; dilution for western blotting, 1:1000), Dll1 (Ab10554; dilution for western blotting, 1:1000), p-mTOR (Ser2448) (Ab109268; dilution for western blotting, 1:1000) and GFAP (Ab33922; dilution for western blotting, 1:1000) were obtained from Abcam (UK). Antibodies against Akt (CST#4691s; dilution for western blotting, 1:1000), p-Akt (Ser473) (CST#4060s; dilution for western blotting, 1:1000), mTOR (CST#2983s; dilution for western blotting, 1:1000), p62 (CST#88588s; dilution for western blotting, 1:1000; dilution for immunofluorescence analysis, 1:100) and LC3B (CST#3868s; dilution for western blotting, 1:1000; dilution for immunofluorescence analysis, 1:100) were obtained from Cell Signaling Technology (USA). Antibodies against NICD (TA336494; dilution for immunofluorescence analysis, 1:100) and GAPDH (TA309157; dilution for western blotting, 1:1000) was obtained from ZSGB-Bio (China). Antibodies against ATP1A1 (14418-1-AP; dilution for western blotting, 1:1000) was obtained from Protein tech (USA).

### Cell culture

U87-MG glioma cells were obtained from the China Academia Sinica Cell Repository (Shanghai, China) and U251-MG glioma cells were obtained from the American Type Culture Collection (ATCC). Cells were cultured in DMEM medium containing 10% FBS (Gibco, US). After MACS, CD133 + cells were cultured in stem cell medium (DMEM/F12 medium supplemented with 10 ng/ml EGF, 10 ng/ml bFGF, and B27 (1:50, Invitrogen, US)). The neurosphere can be observed at the second day.

### Magnetic-activated cell sorting and flow cytometry analysis

CD133 + glioma cells were collected by CD133 MicroBead Kit (Miltenyi, Germany) following the manufacturer’s protocol. The collected cells were stained by CD133 antibody (Miltenyi, Germany) at 4 °C overnight and then Alexa Fluor 488 conjugate anti-mouse secondary antibody. The percentage of CD133 + cells was analyzed by BD FACS Caliber, Aria III (Tianjin Neurological Institute, Tianjin, China).

### Cell viability assay (CCK-8 assay) and in vitro limiting dilution assay

After sorted by FACS and cultured in stem cell medium, secondary neurospheres of U87 and U251 were digested by trypsin into single-celled configuration. Cells (2 × 10^3^ cells per well) were treated with different doses of AZD8055 and rapamycin for 24, 48, and 72 h, with five replicates for each gradient. Ten microliters of Cell Counting Kit-8 (Dojindo Laboratories, Kumamoto, Japan) was added to each well and incubated for 2 h at 37 ℃. The absorbance at 450 nm was measured on a Synergy 2 microplate reader (BioTek). For in vitro limiting dilution assay, GSCs were implanted into a 96-well plate at a gradient of 5, 10, 20, 50, 100, 200, or 500 cells per well, with five replicates for each gradient. The number of tumorspheres in each well was determined after incubation for 7 days, and the sphere formation efficiency were calculated using the Extreme Limiting Dilution Analysis (http://bioinf.wehi.edu.au/software/elda)^58^.

### Soluble drugs treatment

For AZD8055 (Selleckchem #S1555, US) and rapamycin (Selleckchem #S1039), US) treatment, in Western blot, U87 and U251 initiating cells were treated with AZD8055 (0, 0.1, 0.2, and 0.3 μM) for 48 h or treated with rapamycin (0, 1, 2, and 3 µM) for 24 h. In Immunofluorescence analysis, U87 and U251 initiating cells were treated with AZD8055 (0.3 μM) for 48 h and rapamycin (3 µM) for 24 h. For 3-MA (Selleckchem #S2767, US) treatment, in neurosphere formation assay, cell viability assay and in vitro limiting dilution assay, U87 and U251 initiating cells were treated with 0.5 mM 3-MA.

### Immunofluorescence analysis

For neurospheres immunofluorescence staining, an original method was applied to label proteins in the spheroid. Neurospheres were placed into centrifuge tube (Becton, Dickinson and Company, US). The neurospheres were fixed in 4% paraformaldehyde (Solarbio, China) for 10 min at room temperature. They were permeabilized with 0.2% Triton-X-100 for 15 min, and blocked with 5% BSA in PBS for 20 min at room temperature, and incubated with primary antibodies, such as CD133 antibody, Nestin antibody, Notch1 antibody, NICD antibody, p62 antibody, and LC3B antibody overnight at 4 ℃. Alexa fluor-labeled anti-rabbit or anti-mouse antibodies (Invitrogen, USA, 1:500) were added to the samples. The nuclei were stained with DAPI (Sigma-Aldrich). For cells staining, the method is the same as above. Both neurospheres and cells were observed in glass-bottom culture by Perkinelmer UltraVIEW VOX confocal microscope (Institute of neurology, Tianjin Medical University General Hospital, China).

### Western blot

Cells were lysed in the RIPA buffer (Solarbio, China) with PMSF (1:100, Solarbio, China) followed by extracted the total protein. And the total protein concentration was determined using the BCA Protein Assay Kit (Solarbio, China), according to the manufacturer’s instructions. Samples were analyzed by gel electrophoresis, blotted to PVDF membrane (Millipore, US). The primary antibodies used in this study targeted the following proteins: Notch1, NICD, Hes1, Dll1, Nestin, CD133, GFAP, LC3B, p62, mTOR, p-mTOR (Ser2448), Akt, p-Akt (Ser473), and probed using these primary antibodies followed by the HRP-conjugated goat anti-mouse or rabbit IgG antibodies. GAPDH was utilized as loading control. The membrane was developed using the Luminata Classico Western HRP substrate (Millipore, US). Membrane and cytoplasmic proteins were extracted by Subcellular Protein Fraction Kits (Thermo Scientific, US). Anti-sodium potassium ATP1A1 antibody and anti-GAPDH were utilized as loading control for membrane and cytoplasmic proteins, respectively.

### Neurosphere formation assay

GICs treated with different doses of AZD8055 (0, 0.1, 0.2, and 0.3 μM) and rapamycin (0, 1, 2, and 3 µM), 0.5 mM 3-MA alone and 0.3 μM AZD8055 + 0.5 mM 3-MA combination were plated in 96-well plates at a density of 1000 cells per well with five replicates for each gradient, and neurosphere numbers and sizes were calculated at the seventh day after cell placement^59^.

### Intracranial xenograft model in nude mouse

To evaluate whether AZD8055 and rapamycin inhibits tumor growth in vivo, the U87 neurospheres was selected to establish a nude mouse model. Female BALB/c nude mice (4–5 weeks, 15–17 g) were purchased from the Animal Center of the Cancer Institute at Chinese Academy of Medical Science (Beijing, China). To establish an intracranial model, 5 × 10^4^ U87 initiating cells with a luciferase-encoding lentivirus were injected into the mice (*n* = 5 per group) stereotactically. The mice were divided into three groups randomly. Luciferase-encoding lentivirus were purchased from GeneChem (Shanghai, China). The lentivirus vector is GV260: Ubi-MCS-Luc-IRES-Puromycin. The drugs were dissolved in 20%DMSO, 40PEG-300, and 40% PBS. After 7 days post implantation, the mice were injected intraperitoneally with AZD8055 (10 mg/kg/day), rapamycin (1.5 mg/kg/day) or DMSO 5 days a week during the survival period. Intracranial tumor growth was detected by using BLI on days 7, 14, and 28, using the IVIS Spectrum Live Imaging System (PerkinElmer, Branford, USA). The animal research was performed according to the internationally recognized guidelines and national regulations. Brains were extracted and fixed in 10% formalin and then, embedded in paraffin for HE.

### Statistical analysis

All quantified data represent an average of at least triplicate experiments unless otherwise indicated, and standard deviations were calculated. Statistical analysis was performed with SPSS 16 or GraphPad Prism 6.0 software. The differences between two groups were assessed with a Student’s *t*-tests and differences between multiple groups were assessed using a one-way analysis of variance (ANOVA) test followed by Tukey’s post hoc test. Bar graphs were presented as means ± s.d. or means ± s.e.m., with statistical significance at ^#^*P* = NS, *P < 0.05 or **P < 0.01.

## Electronic supplementary material

Supplementary Figure S1

Supplementary Figure S2
