# Supplementary figures and images for: Autophagy suppresses self-renewal ability and tumorigenicity of glioma-initiating cells and promotes Notch1 degradation
Source: Cell Death Dis. 2018 Oct 18;9(11):1063. doi: 10.1038/s41419-018-0957-3 (PMC6194143; doi:10.1038/s41419-018-0957-3)

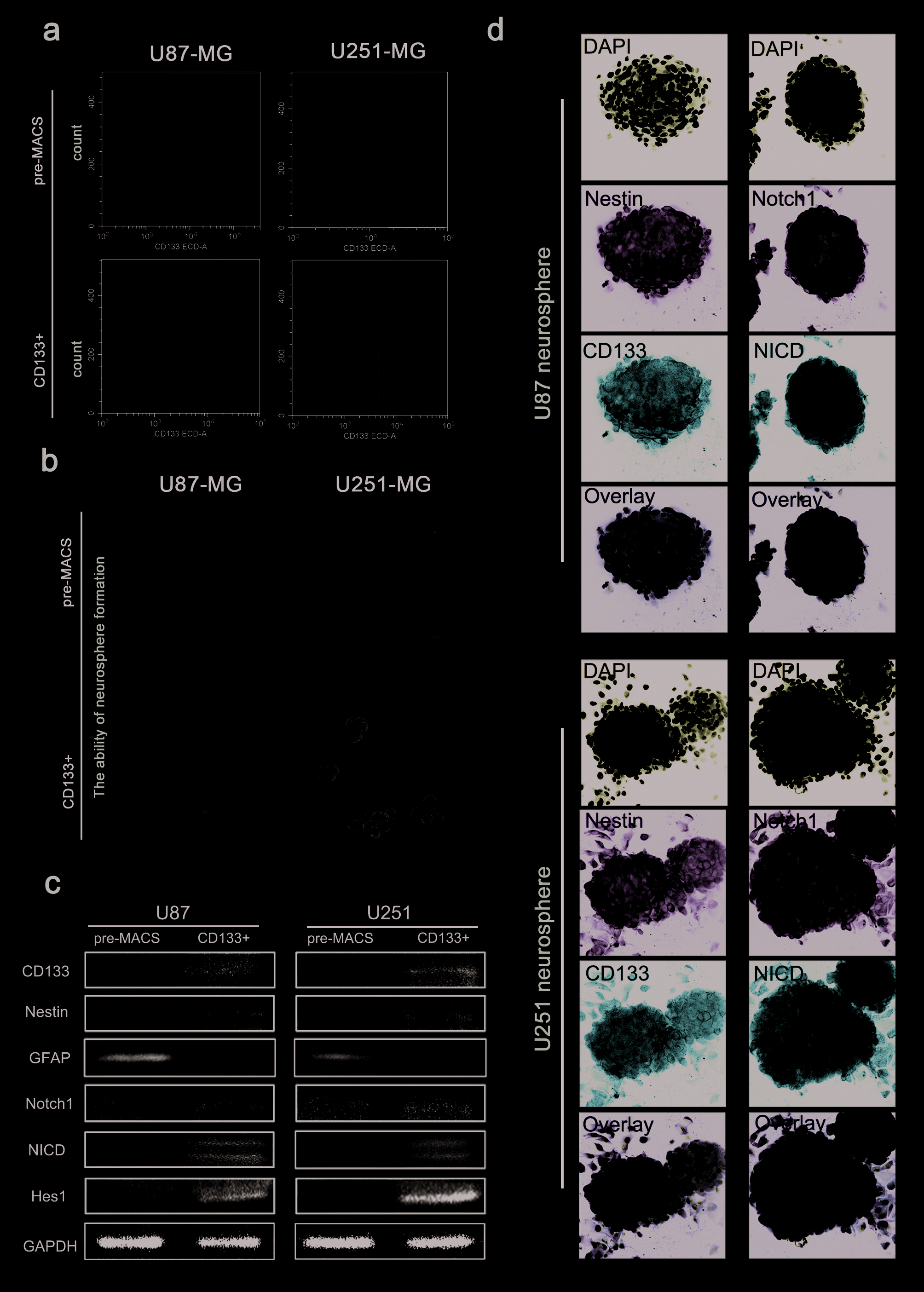

Supplement: Supplementary file 1 — Supplementary Figure S1 [file 41419_2018_957_MOESM1_ESM.doc]

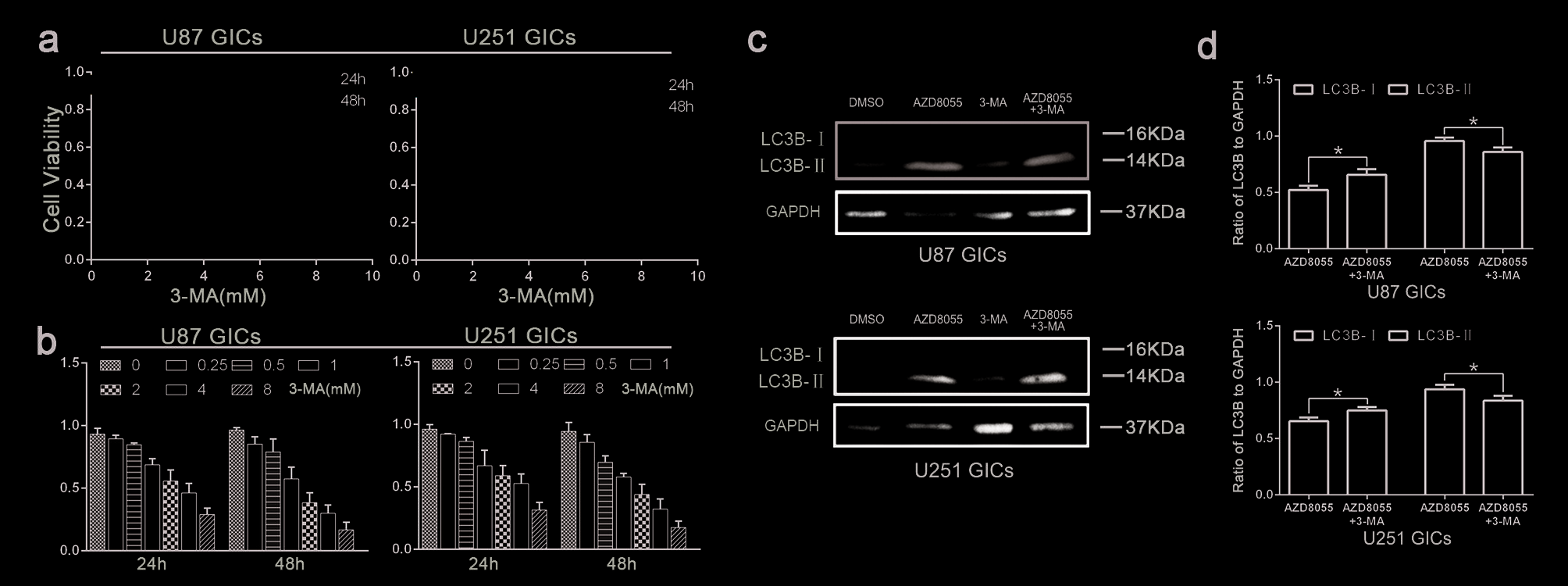

Supplement: Supplementary file 2 — Supplementary Figure S2 [file 41419_2018_957_MOESM2_ESM.doc]
